# Platelet-to-Lymphocyte Ratio and Neutrophil-to-Lymphocyte Ratio as Biomarkers for the Prediction of Activity and Recurrence of Hepatic Echinococcosis: A Retrospective Observational Cohort Study

**DOI:** 10.7759/cureus.96274

**Published:** 2025-11-07

**Authors:** Kamal Al-Jawdah, Karam Mohalab Yaseen, Ghassan Hamdi Hussein

**Affiliations:** 1 General Surgery, Pilgrim Hospital Boston, Lincolnshire, GBR; 2 Department of Surgery, Al-Kindi Teaching Hospital, Baghdad, IRQ

**Keywords:** biomarkers, echinococcosis, hepatic hydatid cyst, neutrophil-to-lymphocyte ratio, platelet-to-lymphocyte ratio, recurrence

## Abstract

Background: Hydatid disease remains a significant health burden in endemic regions, and recurrence after treatment is a major challenge. Platelet-to-lymphocyte ratio (PLR) and neutrophil-to-lymphocyte ratio (NLR) are emerging inflammatory biomarkers, but their role in predicting disease activity and recurrence in hepatic echinococcosis has not been clearly defined.

Objective: This study aimed to evaluate the diagnostic value of PLR and NLR in determining disease activity and predicting recurrence of hepatic hydatid cysts.

Methods: This retrospective observational cohort study was conducted at Al-Kindi Teaching Hospital, Baghdad, and included 187 patients diagnosed with hepatic hydatid cyst (2016-2020) and 190 age-, sex-, and BMI-matched healthy subjects. Only isolated hepatic cases were included, while patients with concomitant organ involvement, ruptured cysts, comorbid diseases, or incomplete records were excluded. Cysts were classified according to the WHO 2001 classification into active (CE1-CE2), transitional (CE3), and inactive (CE4-CE5) stages. All patients were followed for two years post-treatment with serology and ultrasound every six months to detect recurrence, confirmed by CT when indicated. Complete blood counts were obtained at presentation to calculate platelet-to-lymphocyte (PLR) and neutrophil-to-lymphocyte (NLR) ratios.

Results: PLR and NLR were significantly higher among HC patients compared to healthy individuals (p < 0.001). Both markers increased with disease activity, reaching their highest levels in active cysts (PLR median 246.1 vs. 92.8 in inactive, p < 0.0001). During follow-up, recurrence occurred in 27.2% of patients. Recurrent cases showed lower lymphocyte counts and higher neutrophil, platelet, PLR, and NLR values. On multivariate analysis, PLR emerged as the only independent predictor of recurrence (p = 0.033). ROC analysis demonstrated strong discriminative ability for disease activity (PLR AUC = 0.915; NLR AUC = 0.860) and recurrence (PLR AUC = 0.869; NLR AUC = 0.812).

Conclusion: PLR and NLR are significantly elevated in hepatic hydatid cysts, especially in active stages. PLR independently predicts recurrence, highlighting the role of systemic inflammation in cyst persistence. Further studies should validate its prognostic value and optimal cutoffs.

## Introduction

The parasitic infection known as echinococcosis, also known as hydatid disease, is brought on by the larvae of the *Echinococcus* tapeworm genus. Carnivores are the ultimate hosts, whilst humans and herbivores are the accidental and intermediate hosts [1]. It is endemic in the Mediterranean region, Eastern Europe, North and East Africa, South America, Central Asia, China, Australia, and other regions [2]. This parasite could affect different body sites or organs, the liver being the most common organ affected by hydatid cyst (HC) [3].

The majority of patients continue to be asymptomatic or have non-specific symptoms years before the diagnosis. Generally, symptomatic cases present with right upper abdominal pain; in some instances, they present as cases of jaundice due to either compression or invasion of the biliary system, while emergency presentation could be due to the development of complications, as in the case of rupture, which presented with sudden severe abdominal pain or as an anaphylactic reaction [4]. Although it could go unnoticed and be diagnosed in an autopsy, it could present as a serious and life-threatening disease (anaphylactic reaction to rupture cyst is a good example).

According to the causative organism, HC could be divided into hepatic cystic echinococcosis caused by *Echinococcus granulosus*, and the hepatic alveolar echinococcosis (HAE) caused by *Echinococcus multilocularis* [2]. Although HAE's growth has a more indolent course than HCE's, it is more aggressive, with a higher rate of invasion into adjacent organs. HAE has a prognosis comparable to that of a liver tumor, with a 90% mortality rate in 10-15 years after diagnosis in untreated cases [5].

Surgery is the main treatment for the eradication of hydatid disease, with the surgical approach being tailored to each patient depending on the disease activity and the cyst's location in the liver [6]. Depending on the cyst's stage, the WHO 2001 classification categorizes hepatic HCs into five stages of cystic echinococcosis (CE) 1-5, with the choice of management being tailored accordingly [7]. There is a higher chance of complications such as rupture, infection, or compression of nearby structures during the active stages CE1 and CE2 [8].

The choice of method of treatment (antiparasitic medication, percutaneous drainage, or surgery) is largely affected by the HC disease activity [8,9].

The HC can flourish in the liver by mechanisms that enable it to evade the immune system. The chronicity of HC was found to be related to the cluster of differentiation 4 positive (CD4+) helper T cell activity. The ability of the parasite to shift Th1 to Th2 reactivity by inducing certain cytokine responses renders the body less reactive to HC disease [10]. This type of shift was found to be closely related to platelet function [11].

The ratios of platelet-to-lymphocyte (PLR) and neutrophil-to-lymphocyte (NLR) are widely used markers that are obtained from a standard complete blood count (CBC) test. PLR and NLR have gained interest as potential prognostic markers for various conditions. These ratios were thought to be related to systemic inflammatory response, which plays a key role in the pathogenesis of inflammatory [12], infectious [13], and chronic diseases [14], along with several types of cancer [15]. As these ratios are markers for platelet activation and aggregation [16], in addition to inflammation [17], these could explain the proposed mechanism that these markers are able to identify disease activity of HC.

Previous studies explored NLR/PLR in infection and malignancy, but none assessed their role in detecting hepatic echinococcosis recurrence. The possibility of using these markers in cases of HC disease could improve the choice of treatment modality and could affect the patient's outcome.

The aim of the study is to determine the ability of PLR and NLR to be markers for hepatic HC disease activity and recurrence.

## Materials and methods

This is a retrospective observational cohort study that was conducted in Al-Kindi Teaching Hospital (Iraq-Baghdad, Al-Nahda Crossroad). The medical reports of patients diagnosed with hepatic HC during the period extended from January 2016 to January 2020 were included in the study as the HC group, and follow-up of recurrence was continued through the register to January 2022.

The HC group includes patients with a diagnosis of HC of the liver (isolated liver involvement) diagnosed by ultrasound or CT scan with no other medical comorbidities who underwent surgical treatment in our hospital during the data collection period. This was represented in a total of 187 cases.

The healthy group included 190 healthy individuals whose data were obtained from hospital records of routine medical examinations performed for marriage or employment applications. As a part of these health checks, all individuals underwent clinical evaluation and basic blood investigations, including complete blood count. Individuals were included only if they had no history or clinical evidence of hydatid disease, infection, inflammatory, or chronic medical conditions. They were matched to the HC group by age, sex, and body mass index (BMI).

The inclusion criteria were patients with isolated hepatic HCs.

The exclusion criteria were cases of concomitant hydatid disease of other organs (e.g., lung, brain, or other organs), ruptured HC, cases with incomplete reports regarding assessment for recurrence, cases with concomitant medical diseases, and drug or substance abusers.

The data collected included patients' demographics (age, sex, and BMI), the presenting symptoms (pain, jaundice, incidental finding), investigations including CBC at the time of presentation, and imaging (ultrasound or CT scan) with classification of cyst according to the WHO 2001 classification [18]. All cases were followed through the register to identify reporting of recurrence and the need for intervention for the recurrent disease.

Recurrence is defined as the development of new active cysts following therapy; this can include the development of new, distant disease due to spilling or the reappearance of previously treated cysts at the same previous location as a result of continued growth of live cysts [19].

Assessment for recurrence was obtained from the register. The standard protocol of our institute is two years of follow-up of cases by performing serology testing along with ultrasound every six months after surgery or any time symptoms develop. Suspected recurrence confirmed by performing a CT scan. The decision of the secondary intervention is subject to multidisciplinary team discussions.

The study protocol was approved by both scientific and ethical committees of Al-Kindi Teaching Hospital (protocol number 2421A on March 12, 2022). Patient consent was waived as this study was a retrospective one.

Handling of missing data

During follow-up, 73 out of 187 (39%) patients were lost to follow-up before completing the two-year surveillance period. The main reasons for loss included change of residence, inability to attend follow-up appointments due to financial or logistical constraints, and incomplete hospital records during the study period. These cases were excluded from the recurrence analysis but retained in baseline descriptive statistics to preserve representativeness. We performed comparisons between patients with and without follow-up and found no significant differences in baseline demographics or hematological parameters, reducing the likelihood of selection bias. However, this degree of attrition may have reduced statistical power for recurrence-related outcomes and should be considered a potential source of bias.

Statistical analysis

The data were collected and introduced into IBM SPSS Statistics for Windows, version 26.0 (released 2018, IBM Corp., Armonk, NY), and data were presented in the form of tables or charts when appropriate. The normality of the variables was checked using the Shapiro-Wilk test, and the level of significance was checked using chi-square and Fisher's exact tests for categorical data and the Mann-Whitney test for continuous data with two groups, as the variables were non-normally distributed. For continuous variables with three groups Kruskal-Wallis H test, followed by pairwise Mann-Whitney U tests with Bonferroni correction, was used due to the non-normal distribution of the variables. Prediction of recurrences was checked using multivariate analysis using binary logistic regression analysis, and receiver operator curve analysis was used to calculate the area under the curve for the prediction of disease activity and recurrence. A p-value of ≤0.05 is considered statistically significant. ROC curve analysis was used to assess the diagnostic performance of PLR and NLR in differentiating active (CE1-CE2) from inactive (CE4-CE5) hepatic hydatid cysts, as well as in predicting recurrence. The area under the curve (AUC) was calculated for each marker.

## Results

**Figure 1:**
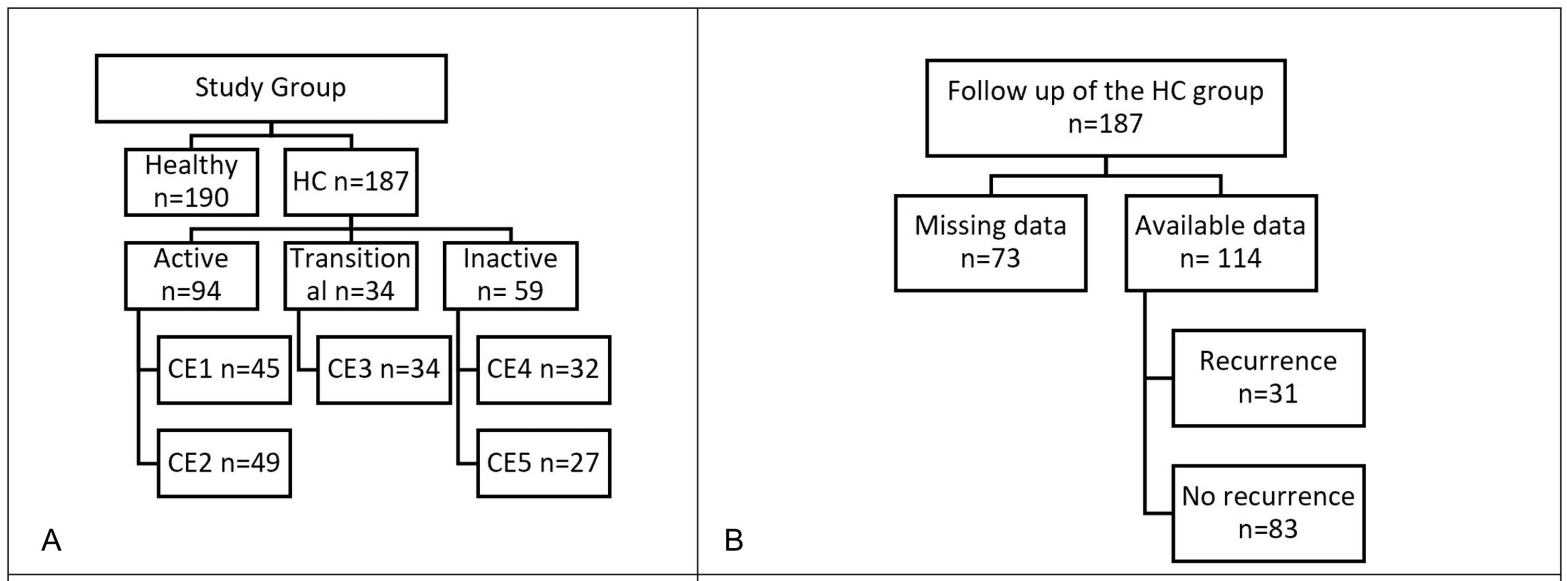
Distribution of the cases and the outcome of the follow-up. A: Distribution of the HC and healthy groups. B: Follow-up of the HC group showing the number of patients with complete and incomplete follow-up and recurrence outcomes. The healthy group underwent baseline evaluation only (no follow-up). HC: hepatic hydatid cyst; WHO: World Health Organization; CE: cystic echinococcosis; BMI: body mass index Figure created using Microsoft Word (Microsoft Corporation, Redmond, WA, USA).

The study included 187 cases of hepatic HC and 190 cases of healthy individuals. The HC group further divided according to the WHO classification of HC into active form (94; 50.3%) cases (which were further divided into CE1 (45; 24.1%) cases and CE2 (49; 26.2%) cases), transitional form (CE3) (34; 18.2%) cases, and inactive form (59; 31.6%) cases (which was further divided into CE4 (32; 17.1%) cases and CE5 (27; 14.4%) cases). The follow-up of the HC group showed that 73 (39%) cases had incomplete follow-up, and 114 (61%) cases completed two years of follow-up. Recurrence was detected in 31 (27.2%) cases, and no recurrence was found in 83 (72.8%) cases, as shown in Figure 1.

The mean age, sex, and BMI level were not different between the HC and healthy groups. The parameters of CBC, apart from total leucocyte count, were different between the two groups. The lymphocyte count was lower in cases of HC than in healthy individuals, while neutrophile and platelet counts, NLR, and PLR were higher than healthy individuals, as described in Table 1.

**Table 1:** Distribution of demographics and investigations between the two groups. * Statistically significant (P value < 0.05 considered statistically significant). † Data presented in the form of number and (percent). ‡ Data presented in the form of median and (interquartile range). BMI: body mass index; NLR: neutrophile-to-lymphocyte ratio; PLR: platelet-to-lymphocyte ratio

Variables		HC n = 187	Healthy n = 190	P-value	Statistical value
Age^‡^ (years)		49 (41-57)	48.5 (41-56)	0.934	Mann-Whitney U Z = -0.083
Sex^†^	Male	59 (31.6)	62 (32.6)	0.822	Chi-square X^2^ = 0.051
	Female	128 (68.4)	128 (67.4)		
BMI (kg/m^2^)^‡^		30.3 (27.2-34)	29.7 (26.2-33.4)	0.093	Mann-Whitney U Z = -1.682
Leucocyte (x10^3^/µL)^‡^		8.78 (7.43-10.19)	8.8 (7.01-10.49)	0.704	Mann-Whitney U Z = -0.377
Lymphocyte (x10^3^/µL)^‡^		1.79 (1.3-2.77)	3.81 (2.65-4.95)	<0.0001^*^	Mann-Whitney U Z = -10.927
Neutrophil (x10^3^/µL)^‡^		3.84 (2.6-5.08)	2.76 (1.66-4.34)	<0.0001^*^	Mann-Whitney U Z = -4.823
Platelet (x10^3^/µL)^‡^		320 (281-364)	286 (230-330)	<0.0001^*^	Mann-Whitney U Z = -6.176
NLR^‡^		2.31 (1.09-3.52)	0.8 (0.39-1.16)	<0.0001^*^	Mann-Whitney U Z = -10.097
PLR^‡^		185.44 (104.12-260.34)	76.62 (54.23-107.92)	<0.0001^*^	Mann-Whitney U Z = -11.165

The comparison of the HC group according to the state of activity of HC is shown in Table 2. Total leukocyte count was not different regarding disease activity. Lymphocyte, neutrophil, platelet counts, NLR, and PLR were significantly higher in cases of active disease than in both transitional and inactive disease. On comparison of the transitional form versus the inactive form, the lymphocyte count and PLR were significantly higher in the transitional form than inactive form. While the remaining investigations were not different between these two forms, as shown in Table 2.

**Table 2:** Distribution of investigation according to disease activity in the HC group. * Statistically significant (P-value < 0.05 considered statistically significant). † P1: active vs. transitional; P2: active vs. inactive; P3: traditional vs. inactive. ‡ Kruskal-Wallis for the initial assessment and Mann-Whitney U test used for the post-hoc analysis. § Active: represent CE1 and CE2 WHO classes, Transitional: represent CE3, and Inactive: represent CE4 and CE5. IQR: interquartile range; NLR: neutrophile-to-lymphocyte ratio; PLR: platelet-to-lymphocyte ratio

Variables	WHO classification^§^			P value^†‡^			
	Active (n = 94)	Transitional (n = 34)	Inactive (n = 59)				
	Median (IQR)	Median (IQR)	Median (IQR)	Overall p-value	P1	P2	P3
Leucocyte (x10^3^/µL)	8.93 (7.79-10.63)	8.51 (7-10.18)	8.59 (7.37-9.98)	P = 0.571 H = 1.607	P = 0.385 Z = -0.869	P = 0.419 Z = -0.808	0.693
Lymphocyte (x10^3^/µL)	1.48 (0.99-1.78)	2.47 (1.42-3.29)	3.02 (2.24-3.95)	P < 0.0001^*^ H = 24.506	P < 0.0001^*^ Z = -4.95	P < 0.0001^*^ Z = -8.562	P = 0.013^*^ Z = -2.473
Neutrophil (x10^3^/µL)	4.49 (3.44-5.45)	3.2 (2-4.14)	3.3 (1.75-4.78)	P < 0.0001^*^ H = 11.825	P = 0.001^*^ Z = -3.299	P < 0.0001^*^ Z = -4.069	P = 0.936 Z = -0.08
Platelet (x10^3^/µL)	359 (320-388)	277.5 (251-332)	285 (261-320)	P < 0.0001^*^ H = 43.054	P < 0.0001^*^ Z = -6.377	P < 0.0001^*^ Z = -7.677	P = 0.582 Z = -0.55
NLR	3.18 (2.38-4.25)	1.17 (0.75-3.01)	1.04 (0.57-1.7)	P < 0.0001^*^ H = 38.977	P < 0.0001^*^ Z = -4.92	P < 0.0001^*^ Z = -8.393	P = 0.208 Z = -1.26
PLR	246.1 (194.03-345)	118.89 (83.84-215.84)	92.83 (72.34-131.3)	P < 0.0001^*^ H = 24.848	P < 0.0001^*^ Z = -6.226	P < 0.0001^*^ Z = -9.277	P = 0.036^*^ Z = -2.098

The assessment of recurrence cases based on the initial investigations showed that, in the WHO classification, the lymphocyte count was lower in the cases of recurrence. Meanwhile, the neutrophil and platelet counts, along with the NLR and PLR, were significantly higher in those who developed recurrence. After the application of multivariate analysis, the PLR was found to be the single independent predictor of recurrence, as shown in Table 3.

**Table 3:** Predictors of recurrence. The dependent variable is recurrence, while the independent variables included in the multivariate model are age, BMI, WHO classification, leukocyte count, neutrophil count, platelet count, NLR, and PLR. Lymphocyte count was excluded due to collinearity. * Statistically significant (P-value <0.05 considered statistically significant). ** Data presented in the form of median and (interquartile range). † Data presented in the form of number and (percent). ‡Multivariate analysis was omitted due to collinearity of the variables. BMI: body mass index; NLR: neutrophile-to-lymphocyte ratio; PLR: platelet-to-lymphocyte ratio

Variables		Recurrence n = 31	No recurrence n = 83	Univariate		Multivariate	
				P-value	Statistical value	P-value	B
Age^**^ (years)		48 (37-55)	52 (43-58)	0.172	Mann-Whitney U Z = -1.367	0.133	0.043
BMI^**^		30.3 (26.5-34)	30.4 (28.1-34.4)	0.741	Mann-Whitney U Z = -0.331	0.645	-0.029
WHO classification^†^	Active	25 (80.6)	43 (51.8)	0.015^*^	Fisher's exact test value = 8.495	0.434	0.386
	Transitional	3 (9.7)	12 (14.5)				
	Inactive	3 (9.7)	28 (33.7)				
Leucocyte (x10^3^/µL)^**^		8.66 (7.8-10.1)	8.81 (7.51-10.15)	0.774	Mann-Whitney U Z = -0.287	0.245	0.203
Lymphocyte (x10^3^/µL)^**‡^		1.24 (0.86-1.46)	2.03 (1.57-2.94)	<0.0001^*^	Mann-Whitney U Z = -6.038	-	-
Neutrophil (x10^3^/µL)^**‡^		4.59 (3.84-5.46)	3.76 (2.36-5.05)	0.035^*^	Mann-Whitney U Z = -2.108	-	-
Platelet (x10^3^/µL)^**‡^		348 (314-405)	319 (268-359)	0.002^*^	Mann-Whitney U Z = -3.137	-	-
NLR^**^		3.52 (2.77-5.63)	1.89 (0.92-2.77)	<0.0001^*^	Mann-Whitney U Z = -5.104	0.059	-0.476
PLR^**^		277.19 (235.48-398.84)	157.62 (101.97-220.96)	<0.0001^*^	Mann-Whitney U Z = -6.040	0.033^*^	-0.008

**Figure 2:**
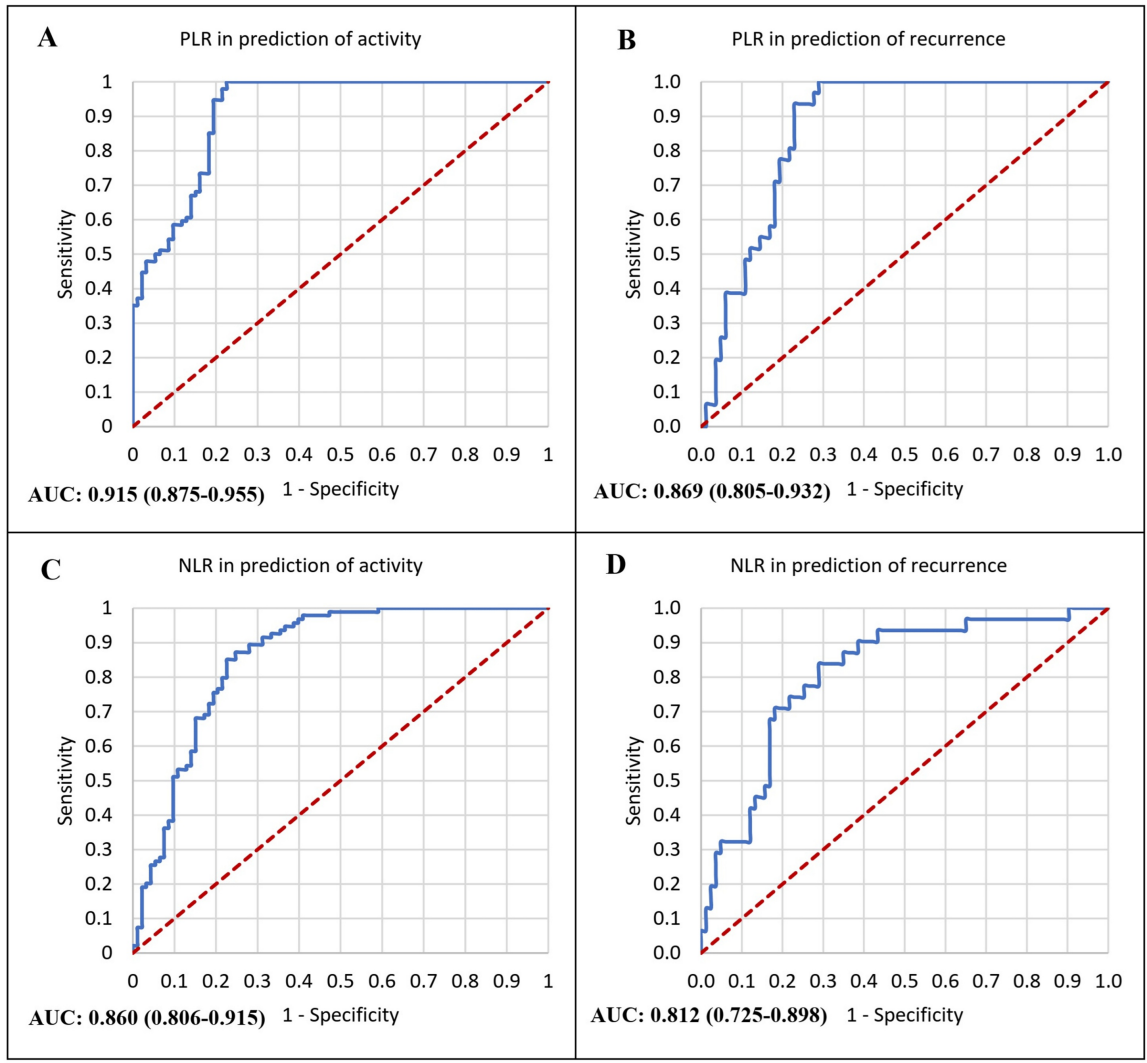
Receiver operator characteristics curve analysis in the prediction of both the activity and recurrence of HC. Prediction of activity refers to the ability of each marker to distinguish active (CE1–CE2) from inactive (CE4–CE5) disease based on the WHO classification. A: ROC curve analysis for PLR in the prediction of HC activity; B: ROC curve analysis for PLR in the prediction of HC recurrence; A: ROC curve analysis for NLR in the prediction of HC activity; B: ROC curve analysis for NLR in the prediction of HC recurrence. * NLR: neutrophile-to-lymphocyte ratio; PLR: platelet-to-lymphocyte ratio; AUC: area under the curve

Receiver operator characteristic curve analysis was performed to evaluate the diagnostic accuracy of PLR and NLR in predicting both disease activity and recurrence of HC. As shown in Figure 2, PLR demonstrated the highest discriminative ability for detecting active disease, with an AUC of 0.915 (95% CI: 0.875-0.955), followed by NLR with an AUC of 0.860 (95% CI: 0.806-0.915). Similarly, for the prediction of recurrence, PLR achieved an AUC of 0.869 (95% CI: 0.805-0.932), while NLR showed an AUC of 0.812 (95% CI: 0.725-0.898), as shown in Figure 2.

## Discussion

Echinococcosis remains a significant public health challenge in many endemic regions, with hepatic HCs carrying the risk of serious complications and recurrence even after treatment. However, reliable biomarkers that predict which cases remain active or recur are scarce, and clinicians currently rely largely on imaging and serology. In a variety of diseases, hematologic ratios (NLR and PLR) have been validated as inexpensive, widely available indicators of systemic inflammation and prognosis [12], and in echinococcosis, preliminary work has shown elevated PLR and NLR in hydatid patients versus healthy individuals [2]. The current study seeks to clarify whether NLR and PLR not only distinguish hydatid patients from healthy individuals but also discriminate disease activity status and predict recurrence risk in hepatic HCs.

The current study showed that HCs were most frequently diagnosed in their active stages (50.3%), followed by inactive (31.6%) and transitional forms (18.2%), according to the WHO classification. This pattern of presentation in this endemic area with a higher rate of active disease is attributed to earlier symptomatic presentation of the disease [4,8].

The recurrence rate in the current study (27.2%) over two years of follow-up is relatively high compared with previous studies; the recurrence rate typically ranges between 4.6% and 22% depending on the surgical technique and follow-up duration [19]. The higher rate in our study may reflect differences in the location of primary HC, as Prousalidis et al.'s [19] study included the recurrence in all body parts, while in the current study, specific hepatic recurrence was found to be higher.

Demographic variables, including age, sex, and BMI, were comparable between HC and healthy controls, indicating that differences in hematologic indices were not confounded by these factors. Lymphocyte counts were markedly reduced, while neutrophil and platelet counts, as well as NLR and PLR, were significantly higher among HC cases. These findings suggest the presence of a systemic inflammatory and immune-modulatory response during hepatic echinococcosis. Similar results were reported by We et al. [2], who found elevated NLR and PLR in both cystic and alveolar echinococcosis compared with healthy individuals, reflecting chronic immune stimulation and altered host inflammatory balance.

The reduction in lymphocyte count in our cohort may indicate immune suppression driven by parasite-induced Th2 polarization, a mechanism previously demonstrated in both experimental and clinical hydatid disease [10]. Elevated neutrophil counts reflect the innate inflammatory reaction against parasitic antigens, while thrombocytosis and increased PLR could be related to platelet activation and cytokine release that promote cyst growth and immune evasion [11].

The current study demonstrated that hematologic markers varied significantly according to the activity stage of HC. While total leukocyte counts did not differ between stages, active cysts (CE1-CE2) were characterized by higher neutrophil and platelet counts and markedly lower lymphocyte counts compared with transitional and inactive forms. Consequently, both NLR and PLR were significantly elevated in active disease, with PLR showing the strongest discriminative ability (AUC = 0.915) for detecting activity. These findings indicate that as the parasite remains viable and metabolically active, it stimulates a sustained inflammatory response and immune modulation, reflected in these blood-derived ratios.

Comparable results were reported by We et al. [2], who observed significantly higher PLR and NLR in active echinococcosis compared with inactive lesions, emphasizing that platelet and neutrophil activation parallels cyst viability and antigenic stimulation. The immune mechanism behind these associations may relate to parasite-induced shifts from Th1 to Th2 responses and cytokine-mediated recruitment of neutrophils and platelets, which promote local inflammation and tissue remodeling [10].

In the present study, recurrence of hepatic HC was significantly associated with elevated NLR and PLR, with PLR emerging as the only independent predictor of recurrence (AUC = 0.869, 95% CI: 0.805-0.932) (Figure 2B). This finding underscores a possible link between sustained systemic inflammation and disease relapse. To our knowledge, no previous studies have examined the predictive role of these hematologic ratios in identifying recurrence of hepatic echinococcosis. Most earlier research has focused on surgical or pharmacologic factors influencing relapse rather than on biological indicators measurable before recurrence develops.

Recurrence after surgical treatment of hydatid disease remains a major challenge, with reported rates ranging between 4.6% and 22% depending on operative technique, cyst type, and follow-up duration [19]. Incomplete cyst removal, intraoperative spillage of protoscolices, and inadequate postoperative surveillance have been cited as principal causes. The recurrence rate in our series (27.8%) (Figure 1) is slightly higher than most reports, which may reflect differences in surgical approach, high prevalence of active cysts, or limited follow-up continuity.

From a pathophysiological perspective, the association between high PLR and recurrence may reflect platelet-mediated inflammatory persistence. Activated platelets release growth factors such as platelet-derived growth factor (PDGF) and transforming growth factor beta (TGF-β), promoting fibrosis, microvascular remodeling, and tissue repair processes that could inadvertently favor parasite persistence or regrowth. Simultaneously, the reduced lymphocyte counts observed in recurrent cases suggest impaired adaptive immunity; *Echinococcus granulosus* antigens are known to shift host immunity toward a Th2-dominant profile, reducing the effectiveness of cellular responses against residual cyst elements [10].

Limitations of the study

The retrospective design of this single-institution study may limit the generalizability, so multicentre studies with a prospective design would be more informative. Another limitation is loss to follow-up/incomplete data. About 39% of the initial cases had incomplete follow-up, which may bias recurrence estimates or reduce power in recurrence analyses, as we used ROC curves within our sample. Without validation in other cohorts (due to unavailability), the optimal cutoffs of PLR or NLR may not generalize.

## Conclusions

PLR and NLR were significantly elevated in patients with hepatic HCs, particularly in active disease stages. Among these, PLR proved to be the only independent predictor of recurrence. These findings suggest that systemic inflammation and platelet activity may influence cyst persistence and relapse. Further studies are needed to confirm its prognostic value and define clinical cutoffs.

## References

[1] Gessese AT (2020). Review on epidemiology and public health significance of hydatidosis. Vet Med Int.

[2] E W, Wang Z, Pang M, Lu Y, Fan H (2021). The correlation between platelet-to-lymphocyte ratio and neutrophil-to-lymphocyte ratio with hepatic echinococcosis. J Inflamm Res.

[3] Khalili N, Iranpour P, Khalili N, Haseli S (2023). Hydatid disease: a pictorial review of uncommon locations. Iran J Med Sci.

[4] Faraj W, Abi Faraj C, Kanso M (2022). Hydatid disease of the liver in the Middle East: a single center experience. Surg Infect (Larchmt).

[5] Tamarozzi F, Mariconti M, Covini I, Brunetti E (2017). Rapid diagnostic tests for the serodiagnosis of human cystic echinococcosis [Article in French]. Bull Soc Pathol Exot.

[6] Deo KB, Kumar R, Tiwari G, Kumar H, Verma GR, Singh H (2020). Surgical management of hepatic hydatid cysts - conservative versus radical surgery. HPB (Oxford).

[7] Ferrer Inaebnit E, Molina Romero FX, Segura Sampedro JJ, González Argenté X, Morón Canis JM (2022). A review of the diagnosis and management of liver hydatid cyst. Rev Esp Enferm Dig.

[8] Paramita AAKY, Wibawa IDN (2023). Multimodal Treatment of Cystic Echinococcosis. Indones J Gastroenterol Hepatol Dig Endosc.

[9] Botezatu C, Mastalier B, Patrascu T (2018). Hepatic hydatid cyst - diagnose and treatment algorithm. J Med Life.

[10] Rostami-Rad S, Jafari R, Yousofi Darani H (2018). Th1/Th2-type cytokine profile in C57 black mice inoculated with live Echinococcus granulosus protoscolices. J Infect Public Health.

[11] Li Q, Liu Y, Wang X (2021). Regulation of Th1/Th2 and Th17/Treg by pDC/mDC imbalance in primary immune thrombocytopenia. Exp Biol Med (Maywood).

[12] Al-Jawdah K, Kamal Z (2022). Complete blood count parameters in the diagnosis of acute appendicitis. J Pharm Negat Results.

[13] Russell CD, Parajuli A, Gale HJ (2019). The utility of peripheral blood leucocyte ratios as biomarkers in infectious diseases: a systematic review and meta-analysis. J Infect.

[14] Tudurachi BS, Anghel L, Tudurachi A, Sascău RA, Stătescu C (2023). Assessment of inflammatory hematological ratios (NLR, PLR, MLR, LMR and monocyte/HDL-cholesterol ratio) in acute myocardial infarction and particularities in young patients. Int J Mol Sci.

[15] Fang T, Wang Y, Yin X (2020). Diagnostic sensitivity of NLR and PLR in early diagnosis of gastric cancer. J Immunol Res.

[16] Artoni A, Abbattista M, Bucciarelli P (2018). Platelet to lymphocyte ratio and neutrophil to lymphocyte ratio as risk factors for venous thrombosis. Clin Appl Thromb Hemost.

[17] Wang RH, Wen WX, Jiang ZP (2023). The clinical value of neutrophil-to-lymphocyte ratio (NLR), systemic immune-inflammation index (SII), platelet-to-lymphocyte ratio (PLR) and systemic inflammation response index (SIRI) for predicting the occurrence and severity of pneumonia in patients with intracerebral hemorrhage. Front Immunol.

[18] WHO Informal Working Group (2003). International classification of ultrasound images in cystic echinococcosis for application in clinical and field epidemiological settings. Acta Tropica.

[19] Prousalidis J, Kosmidis C, Anthimidis G, Kapoutzis K, Karamanlis E, Fachantidis E (2012). Postoperative recurrence of cystic hydatidosis. Can J Surg.

